# Early diagnosis of mild cognitive impairment and mild dementia through basic and instrumental activities of daily living: Development of a new evaluation tool

**DOI:** 10.1371/journal.pmed.1002250

**Published:** 2017-03-14

**Authors:** Elise Cornelis, Ellen Gorus, Ingo Beyer, Ivan Bautmans, Patricia De Vriendt

**Affiliations:** 1 Department of Geriatrics, Universitair Ziekenhuis Brussel, Brussels, Belgium; 2 Frailty in Ageing Research Group (FRIA), Vrije Universiteit Brussel, Brussels, Belgium; 3 Department of Occupational Therapy, Artevelde University College Ghent, Ghent, Belgium; 4 Department of Gerontology (GERO), Vrije Universiteit Brussel, Brussels, Belgium; University of Cambridge, UNITED KINGDOM

## Abstract

**Background:**

Assessment of activities of daily living (ADL) is paramount to determine impairment in everyday functioning and to ensure accurate early diagnosis of neurocognitive disorders. Unfortunately, most common ADL tools are limited in their use in a diagnostic process. This study developed a new evaluation by adopting the items of the Katz Index (basic [b-] ADL) and Lawton Scale (instrumental [i-] ADL), defining them with the terminology of the International Classification of Human Functioning, Disability and Health (ICF), adding the scoring system of the ICF, and adding the possibility to identify underlying causes of limitations in ADL.

**Methods and findings:**

The construct validity, interrater reliability, and discriminative validity of this new evaluation were determined. From 2015 until 2016, older persons (65–93 y) with normal cognitive ageing (healthy comparison [HC]) (*n* = 79), mild cognitive impairment (MCI) (*n* = 73), and Alzheimer disease (AD) (*n* = 71) underwent a diagnostic procedure for neurocognitive disorders at the geriatric day hospital of the Universitair Ziekenhuis Brussel (Brussels, Belgium). Additionally, the ICF-based evaluation for b- and i-ADL was carried out. A global disability index (DI), a cognitive DI (CDI), and a physical DI (PDI) were calculated. The i-ADL-CDI showed high accuracy and higher discriminative power than the Lawton Scale in differentiating HC and MCI (area under the curve [AUC] = 0.895, 95% CI .840–.950, *p* = .002), MCI and AD (AUC = 0.805, 95% CI .805–.734, *p* = .010), and HC and AD (AUC = 0.990, 95% CI .978–1.000, *p* < .001). The b-ADL-DI showed significantly better discriminative accuracy than the Katz Index in differentiating HC and AD (AUC = 0.828, 95% CI .759–.897, *p* = .039). This study was conducted in a clinically relevant sample. However, heterogeneity between HC, MCI, and AD and the use of different methods of reporting ADL might limit this study.

**Conclusions:**

This evaluation of b- and i-ADL can contribute to the diagnostic differentiation between cognitively healthy ageing and neurocognitive disorders in older age. This evaluation provides more clarity and nuance in assessing everyday functioning by using an ICF-based terminology and scoring system. Also, the possibility to take underlying causes of limitations into account seems to be valuable since it is crucial to determine the extent to which cognitive decline is responsible for functional impairment in diagnosing neurocognitive disorders. Though further prospective validation is still required, the i-ADL-CDI might be useful in clinical practice since it identifies impairment in i-ADL exclusively because of cognitive limitations.

## Abstract 2

Elise Cornelis and colleagues describe the development of a new evaluation tool for early diagnosis of mild cognitive impairment and mild dementia through basic and instrumental activities of daily living.

## Author summary

**Why was this study done?:**

- Mild cognitive impairment (MCI) is seen as a transitional zone between normal aging and dementia.
- Assessment of activities of daily living (ADL) is paramount to underpin accurate diagnostic classification in MCI and dementia.
- Unfortunately, most common report-based ADL tools have limitations for diagnostic purposes.
- We set out to develop and validate a new tool to evaluate basic (b-; activities including personal hygiene, dressing, and eating) and instrumental (i-; cooking, shopping, and managing medication) ADL in an older population with cognitive disorders.

**What did the researchers do and find?:**

- We developed a tool based on the framework of the International Classification of Functioning, Disability and Health (ICF).
- A global disability index (DI), a Cognitive DI (CDI) (a disability index taking into account solely activities impaired because of cognitive reasons), and a Physical DI (PDI) (a disability index taking into account solely activities impaired because of physical reasons) were calculated for both b- and i-ADL, based on the number of activities performed and the severity and causes of the functional problem.
- 223 community-dwelling older persons diagnosed as (1) cognitively healthy participants (*n* = 79), (2) patients with MCI (*n* = 73), or (3) mild to moderate dementia (*n* = 71) were included and underwent the new ICF-based evaluation of b- and i-ADL.
- The i-ADL-CDI showed high accuracy and discriminative power in differentiating healthy comparisons (HC), MCI, and AD, and the b-ADL-DI showed high discriminative accuracy in differentiating HC and AD.

**What do these findings mean?:**

- The new ICF-based evaluation of b- and i-ADL showed a good ability to distinguish normal and pathological cognitive aging and might enable the diagnosis of MCI and mild dementia.
- The discriminative power of the ICF-based evaluation of b- and i-ADL for underlying causes of limitations is an advantage.
- Since an evaluation of ADL is experienced as less invasive by older persons, this evaluation offers directions for clinical use in diagnosing cognitive disorders and might offer possibilities for clinical treatment, rehabilitation, advising, and coaching.
- This study was conducted in a clinically relevant sample at a geriatric day hospital. However, heterogeneity in the participants might limit this study.

## Introduction

Health services are dealing with an increasing number of older patients. Although most seniors are in reasonably good health and living an active life, a considerable number of them are at risk of developing major chronic conditions and mental disorders such as dementia. Worldwide, it is estimated that dementia affects 46.8 million persons, which causes great stress to medical, social, and informal care [1,2]. Several interventions have already proven efficient in reducing caregiver strain, psychological morbidity, and delaying or avoiding admissions in residential care. Since such interventions may be more effective early in the disease course, early diagnosis of dementia is pivotal [3,4]. In this regard, the concept of mild cognitive impairment (MCI) is interesting since it is seen as a transitional zone between normal aging and dementia. However, MCI is a heterogeneous concept in its clinical presentation and its progression to dementia; mainly, amnestic MCI (a-MCI) has high risk of dementia, but some persons remain stable or even revert to normal cognition [5–8]. Boundaries between normal aging, MCI, and mild dementia are vague, and discussion about the MCI criteria and their operationalization is ongoing [6,9]. The differentiation between mild and major neurocognitive disorders (NCD)—referring to the new version of the Diagnostic Statistical Manual of Mental Disorders (DSM-5) [10]—may be a step in a good direction since this entails a stronger emphasis on “independence in activities of daily living (ADL)” [11–14]. The distinction between mild and major NCD is determined by the extent to which cognitive decline interferes with everyday functioning [12,15]. In major NCD or dementia, cognitive impairment influences independence in everyday functioning in a negative way. In mild NCD or MCI, individuals remain autonomous [15,16], although subtle problems may already occur in complex activities [12,17–21]. The process of functional decline shows a typical and distinctive progression [22,23]. Instrumental ADL (i-ADL) such as cooking, shopping, and managing medication will become slightly limited in mild NCD and will require support in major NCD [18,23–26]. Basic ADL (b-ADL), which includes personal hygiene, dressing, and eating, remain stable the longest [27]. Only in major NCD does one need the support of others in performing b-ADL [23,28,29]. Consequently, assessment of ADL is paramount to determine the degree of impairment in everyday functioning and to underpin accurate diagnostic classification in NCD [9,12,15,30]. Besides, ADL disability might increase the risk for incident dementia. In that way, an evaluation of ADL might be useful not just as diagnostic tool but also as an indicator of the risk for future dementia [12,30].

In clinical practice, information about ADL is most commonly ascertained by asking a patient or his or her caregiver to report about the everyday functioning [31]. Report-based ADL scales are usually quick and easy to administer [9,32,33]. Unfortunately, most common report-based ADL tools have limitations for diagnostic purposes. Firstly, they often use scoring systems solely assessing the success or failure of completing a task [17,34]. They do not reflect the process of performing the task, although this could be meaningful for diagnostic purposes, particularly in mild cognitive disorders [35–39]. Secondly, evaluations of ADL often entail gender-dependent tasks, tasks that a person does not perform, or tasks that become subject to family support, which commonly comes into play among an older population. Clear-cut guidelines on how to deal with tasks that a person does not perform are lacking [40]. Thirdly, ADL evaluations have a poor sensitivity to detect mild functional impairments and are mostly unresponsive to detect changes in a person’s ability level [41–44]. The discriminative power of existing tools is insufficient, and psychometric properties are either unavailable or do not meet standards of quality [9,43,45]. Finally, assessment tools do not differentiate between underlying causes of limitations [46]. Nevertheless, in diagnosing NCD in a geriatric population, it is crucial to determine the extent to which cognitive decline is responsible for functional impairment, since comorbidities, physical limitations, or other noncognitive causes of decline in ADL are often seen in old age [12,47].

Over the years, multiple report-based ADL scales have been developed in order to contribute to the early diagnosis of NCD [31]. Tools such as the Functional Activities Questionnaire (FAQ) [48], the Everyday Technology Use Questionnaire (ETUQ) [49], and the Everyday Cognition (ECog) [50] have already targeted some shortcomings of current evaluations by including “new” items specific to everyday technologies and using scoring systems that only take activities into account that are relevant to an individual. These evaluations showed promising results in assessing individuals with NCD [31]. However, they do not solely assess performance in b- or i-ADL but rather evaluate a mixed spectrum of self-care, household, and other activities or assess everyday abilities such as memory, language, or divided attention.

To address the concerns of report-based ADL scales, performance-based scales such as the Assessment of Motor and Process Skills [51,52] and the Naturalistic Action Test [53,54] have been developed. These evaluations examine the process of task performance, detect changes in everyday functioning, and address causation in observable behaviors. However, these assessments also have limitations, such as being more time-consuming and needing a high degree of training of the assessors, which often limit its use in clinical practice [9,31]. Furthermore, most performance-based ADL scales are not freely available and are mostly not yet validated for use in MCI [55].

Currently, the most commonly used tools for assessing b- and i-ADL are respectively the Katz Index [22] and the Lawton Scale [56] [31,57,58]. Although in widespread use, both scales have shortcomings as mentioned above: they have poorly described psychometric properties, the scoring systems are not sensitive enough to detect subtle deficits, and they do not identify causes of limitations in ADL [9,43,58–61]. Many studies have attempted to improve the potential use of the Katz Index and Lawton Scale, including using item response theory methods [34], providing short versions of these scales [62], or by combining both scales in new evaluations [63,64]. However, these improvements could not overcome all mentioned shortcomings.

Therefore, this study set out to develop a new tool to evaluate b- and i-ADL for diagnostic purposes in a geriatric population with NCD. This evaluation is based on the International Classification of Functioning, Disability and Health (ICF) developed by the World Health Organization (WHO) [65]. The ICF provides a framework for describing everyday functioning and advances the understanding and measurement of disability [58]. It is increasingly being applied in clinical practice and research and has gained acceptance as the worldwide framework of assessing human functioning [66,67]. The new evaluation adopted the activities of the Katz Index and Lawton Scale—since they are considered sound as items for describing functioning in b- and i-ADL [68]—and were defined with the ICF terminology. Besides, the new evaluation took over the scoring system of the ICF and added the possibility to determine underlying causes of limitations. This evaluation might be useful in clinical and research settings to evaluate everyday functioning in NCD since it has an advantage over currently used report-based scales by applying the ICF terminology and scoring system. This offers a more standardized evaluation of ADL, which might benefit a more reliable and accurate diagnosis and treatment of NCD. In this study, the construct validity, interrater reliability, and discriminative validity of this new evaluation were determined. We hypothesized that the ICF-based evaluation of b- and i-ADL will have a good construct validity and interrater reliability and will be able to discriminate between cognitively healthy comparisons (HC), MCI, and AD.

## Methods

The study protocol was based on the STARD criteria, developed to improve the completeness and transparency of reporting of studies of diagnostic accuracy [69].

### Ethics statement

The Ethical Committee of the Universitair Ziekenhuis Brussel approved this study (B.U.N. 143201523678). All data were collected in accordance with the ICH-GCP guidance and the declaration of Helsinki. All participants and informants gave written informed consents.

### Participants and procedure

Three groups of community-dwelling older persons (≥65 y) were recruited consecutively through the geriatric day hospital of an academic teaching hospital (UZ Brussel, Belgium): (1) HC, (2) patients with MCI, and (3) with Alzheimer disease (AD). Patients with MCI and AD underwent a procedure for the diagnosis of cognitive disorders that was performed by a multidisciplinary team and is considered as good clinical practice [70]. This procedure consisted of a physical and neurological examination, clinical history taking, and neuropsychological assessment using the Mini-Mental State Examination (MMSE) [71]; Cambrigde Examination for mental disorders of the elderly, cognitive part (CamCog) [72]; memory subscale of the Alzheimer’s Disease Assessment Scale [73]; Visual Association Test (VAT) [74]; Memory Impairment Screen (MIS) [75]; Trail Making Test, parts A and B [76,77]; Frontal Assessment Battery [78]; and Geriatric Depression Scale (GDS-15) [79]. The procedure was completed by an evaluation of ADL using the Katz Index and Lawton Scale, extensive laboratory blood testing, and imaging of the brain by CT or MRI scan. HC were recruited separately from the diagnostic process for MCI and AD. They represent a heterogeneous sample of community-dwelling volunteers and geriatric patients who visited the geriatric day hospital for the diagnosis or treatment of conditions other than cognitive disorders (e.g., osteoporosis). HC were evaluated by the researchers using the same neuropsychological assessment and evaluation of ADL as MCI and AD. For all groups, the number of comorbidities and medication use was inventoried. The number of comorbidities was determined by counting the active diseases listed in the medical records at the moment the participants visited the geriatric day hospital, whether they were being treated pharmaceutically or not. All active diseases were counted in HC or as co-occurring with MCI or AD.

#### Cognitively healthy persons

Exclusion criteria for the HC (*n* = 79) were a history of NCD, a score <26/30 on the MMSE, and any self- or informant-based complaint of functional or cognitive deficits, which were suggestive of MCI or AD. Exclusion criteria were a score of <80/105 on the total CamCog score, <18/27 on the memory section of the CamCog score, and <8/12 on the MIS and VAT [80,81].

#### Patients with MCI

Patients with MCI (*n* = 73) were diagnosed by clinical consensus of the multidisciplinary team and fulfilled the diagnostic criteria for a-MCI as defined by Petersen [5]. The presence of a major depression was ruled out prior to the diagnosis of MCI.

#### Patients with AD

Patients with AD (*n* = 71) fulfilled the National Institute for Neurological and Communicative Disorders and Stroke—Alzheimer’s Disease and Related Disorder Association (NINDS-ADRDA) criteria [82]. Decisions on the diagnosis of AD were also based on the results of the diagnostic procedure and were carefully taken by consensus of the multidisciplinary team. When the presence of a major depression was presumed, this was ruled out prior to the diagnosis of AD.

For all participants, exclusion criteria included any acute pathology, sensory, or communicative impairments that precluded them from participating; history of major psychiatric illness; or any pathology of the central nervous system other than MCI or AD (e.g., stroke, epilepsy). An additional exclusion criterion for patients with MCI or AD was the absence of a reliable informant, in order to control over- or underestimation of functional abilities. Informants were considered as reliable when they were spouses, family, or close friends who were able to provide accurate information about the participant’s daily life. The proxy’s ability to provide accurate information was operationalized by asking each person with MCI or AD if the proxy was someone who knew him or her well and could provide accurate information about his or her daily life.

After the procedure, on the same day, trained occupational therapists carried out the new ICF-based evaluation of everyday functioning in b- and i-ADL. When conducting the new evaluation, the occupational therapists were blinded to the results of the other evaluations and the diagnosis.

### The ICF-based evaluation of everyday functioning in b- and i-ADL

This evaluation has been designed as a semistructured interview that takes 10 min to complete. For the HC, self-report was used. For MCI and AD, proxy report was conducted.

### Items according to the ICF definitions

According to the linking rules of Cieza et al. (2005) [83], the content of each item included in the Katz Index [22] and the Lawton Scale [56,84] was linked to one or more definitions of the activities component of the ICF (Tables 1 and 2).

**Table 1:** Items of the Katz Index according to the codes and definitions of the ICF.

Item according to Katz’s Index	ICF activity	ICF code	ICF definition
Bathing	Washing oneself	d510	Washing and drying one’s whole body, or body parts, using water and appropriate cleaning and drying materials or methods, such as bathing, showering, washing hands and feet, face and hair, and drying with a towel.
Dressing	Dressing	d540	Carrying out the coordinated actions and tasks of putting on and taking off clothes and footwear in sequence and in keeping with climatic and social conditions, such as by putting on, adjusting and removing shirts, skirts, blouses, pants, undergarments, saris, kimono, tights, hats, gloves, coats, shoes, boots, sandals and slippers.
Transferring	Indoor mobility and changing basic body position	d410	Getting into and out of a body position and moving from one location to another, such as getting up out of a chair to lie down on a bed, and getting into and out of positions of kneeling or squatting.
	Transferring oneself	d420	Moving from one surface to another, such as sliding along a bench or moving from a bed to a chair, without changing body position.
	Walking	d450	Moving along a surface on foot, step by step, so that one foot is always on the ground, such as when strolling, sauntering, walking forwards, backwards, or sideways.
Continence	Regulating urination	d5300	Coordinating and managing urination, such as by indicating need, getting into the proper position, choosing and getting to an appropriate place for urination, manipulating clothing before and after urination, and cleaning oneself after urination.
	Regulating defecation	d3501	Coordinating and managing defecation, such as by indicating need, getting into the proper position, choosing and getting to an appropriate place for defecation, manipulating clothing before and after defecation, and cleaning onself after defecation
Toileting	Toileting	d530	Planning and carrying out the elimination of human waste (urination and defecation), and cleaning oneself afterwards.
Feeding	Eating	d550	Carrying out the coordinated tasks and actions of eating food that has been served, bringing it to the mouth and consuming it in culturally acceptable ways, cutting or breaking food into pieces, opening bottles and cans, using eating implements, having meals, feasting or dining.
	Drinking	d560	Taking hold of a drink, bringing it to the mouth, and consuming the drink in culturally acceptable ways, mixing, stirring and pouring liquids for drinking, opening bottles and cans, drinking through a straw or drinking running water such as from a tap or a spring.

**Table 2:** Items of the Lawton Scale according to the codes and definitions of the ICF.

Item according to Lawton Scale	ICF activity	ICF code	ICF definition
Telephone use	Using communication devices and techniques	d360	Using devices, techniques and other means for the purposes of communicating, such as calling a friend on the telephone.
Using transportation	Using transportation	d470	Using transportation to move around as a passenger, such as being driven in a car or on a bus, rickshaw, jitney, animal-powered vehicle, or private or public taxi, bus, train, tram, subway, boat or aircraft.
Shopping	Shopping	d6200	Obtaining, in exchange for money, goods and services required for daily living (including instructing and supervising an intermediary to do the shopping), such as selecting food, drink, cleaning materials, household items or clothing in a shop or market; comparing quality and price of the items required, negotiating and paying for selected goods or services, and transporting goods.
Preparing food	Preparing meals	d630	Planning, organising, cooking and serving simple and complex meals for oneself and others, such as by making a menu, selecting edible food and drink, getting together ingredients for preparing meals, cooking with heat and preparing cold foods and drinks, and serving the food.
Housekeeping	Doing housework	d640	Managing a household by cleaning the house, washing clothes, using household appliances, storing food and disposing of garbage, such as by sweeping, mopping, washing counters, walls and other surfaces; collecting and disposing of household garbage; tidying rooms, closets and drawers; collecting, washing, drying, folding and ironing clothes; cleaning footwear; using brooms, brushes and vacuum cleaners; using washing machines, driers and irons.
Doing laundry	Washing and drying clothes	d6400	Washing clothes and garments and hanging them out to dry in the air.
Doing handyman work	Caring for household objects	d650	Maintaining and repairing household and other personal objects, including house and contents, clothes, vehicles and assistive devices, and caring for plants and animals, such as painting or wallpapering rooms, fixing furniture, repairing plumbing, ensuring the proper working order of vehicles, watering plants, grooming and feeding pets and domestic animals.
Responsibility for own medications	Maintaining one’s health	d5702	Caring for oneself by being aware of the need and doing what is required to look after one's health, both to respond to risks to health and to prevent ill-health, such as by seeking professional assistance; following medical and other health advice; and avoiding risks to health such as physical injury, communicable diseases, drug-taking and sexually transmitted diseases.
Handling finance	Basic economic transitions	d860	Engaging in any form of simple economic transaction, such as using money to purchase food or bartering, exchanging goods or services; or saving money.

### Interview protocol

First, the participant or proxy is asked whether an activity was performed during the past years. It is expected that the interviewer uses the ICF definitions to clarify the content of an activity. Each activity is rated for its relevance, which means that it is currently performed or it was previously performed by the individual. If activities have not been carried out during the past years because they were not relevant for an individual, they are not taken into account. This is mainly important for i-ADL, since these activities may never have been performed before (e.g., gender relevant) and are consequently irrelevant for the individual. For b-ADL, all items are relevant for every individual since—according to the definition of Reuben [85]—these activities are necessary to survive. The sum of relevant activities leads to the Total Number of relevant Activities (TNA). There is no cutoff of how many items are allowed to be not relevant.

### Scoring system

The participant or proxy is asked how the activities are currently being performed. Based on the description, the investigator assigns a score. The scoring system adopted the performance qualifiers of the ICF, consisting of a five-point scale ranging from 0 (no difficulty to perform) to 4 (complete difficulty or unable to perform) (Table 3). Each score describes how an activity is performed (ICFScoreAct). The qualifiers were operationalized based on the experience of this research team with the development of the advanced ADL tool (a-ADL tool) [35,86] and on a previous qualitative study [87]. The sum of activities with a limitation (score ≥ 1) leads to the total number of Limited Activities (LimAct).

**Table 3:** Scoring system adopted from the performance qualifiers of the ICF.

ICFScore 0	The activity is carried out completely independently, no help is needed. There are no limitations in performing the activity. It is carried out adequately and in a normal frequency.
ICFScore 1	The activity is carried out completely independently, no help is needed but there are mild limitations. The person performs the activity less frequent, more simplified, more rigid and needs more time to complete. The person is less flexible, inventive and more rigid.
ICFScore 2	Mostly, the activity is carried out independently but sometimes help from others is needed. There are moderate limitations: it is less result oriented, less adequate and there are occasionally errors.
ICFScore 3	The activity is carried out completely dependently. Continuous help from others is needed (guiding, modelling or support). The person experiences severe problems in performing the activity and makes many errors.
ICFScore 4	The activity is no longer carried out. The person stopped performing this activity or is unable to perform the activity. If necessary, it has been taken over by others.

Operationalized by Cornelis et al. [87] and De Vriendt et al. [35,86].

### Causes of limitations

If a score of 1 or higher is assigned, the interviewer determines the underlying cause of limitation by asking the participant what causes the limitations. The interviewer probes with the following questions: “Why do you/does (s)he performs this activity differently?” or “What causes the need for help to perform this activity?” In this way, the interviewer interprets the story of the participant and can distinguish cognitive reasons (e.g., global mental functions, memory, attention, etc.), physical reasons (e.g., sensorial functions, mobility, stability, etc.), intrapersonal reasons (e.g., switch in field of interest), social reasons (e.g., loss of partner), and environmental reasons (e.g., car sold, moving to a new place, etc.) of limitations. The assignment of a reason is dichotomous: “yes” when a reason is present and “no” when a reason is absent. It is possible to assign more than one reason of limitation. To clarify how ICF scores can be derived and physical or cognitive causes of limitations can be assigned, some examples are illustrated in Table 4.

**Table 4:** Examples of ICF scores and physical or cognitive reasons of limitations.

Activity	Example of an answer	ICFScore	Reason of limitation
b-ADL: Dressing	*My father needs more time to dress himself*. *He is slower and it looks like* ***he needs more effort to focus***, *but he is still performing it on his own*.	1	Cognitive
i-ADL: Using transportation	*My mother only uses public transport when she is accompanied by someone else*. ***Without help*, *she wouldn’t be able to get in or out a bus or tram*.**	3	Physical
b-ADL: Regulating urination	*I’ve noticed that my husband is less hygienic when using the toilet*. *Sometimes he urinates a little beside the toilet or has he a few spots of urine on his clothing*. *He seems to be less skillful*, *it is like* ***he doesn’t understand it anymore although he can motorically handle it*.** *Sometimes he needs my help*.	2	Cognitive
i-ADL: Preparing food	*Three times a week*, *I prepare a meal for my mother*. *On the other days she does it by herself but I’ve noticed that* ***it takes a lot of effort for her and that she makes mistakes*.** *Once I saw her boiling potatoes in milk*!	2	Cognitive
b-ADL: Indoor mobility	*My wife spends her day in a wheelchair*. ***She isn’t able to move herself*.** *For transfers*, *she needs complete help from me and a nurse*.	4	Physical
b-ADL: Indoor mobility	*Sometimes*, *my father seems to be* ***disoriented in the house***. *Occasionally it is like he doesn’t understand were to go when I ask him to go to the kitchen*.	2	Cognitive
i-ADL: Basic economic transactions	*I still manage my money on my own*. *But since recently*, *when I need to pay or have to use money*, ***I really need to focus and pay attention***. *I also have some difficulties with* ***reading small numbers*** *and I don’t want to make mistakes*!	1	Cognitive + Physical

### Indices

**Fig 1:**
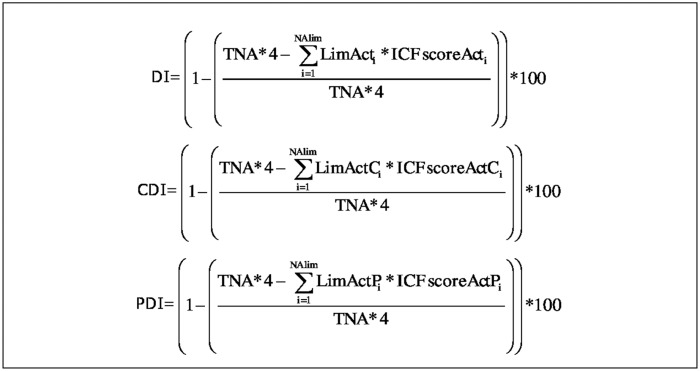
Formulas to calculate the indices of both b- and i-ADL. TNA = Total Number of Activities; LimAct_i_ = the i-th total number of Limited Activities; LimActC_i_ = the i-th total number of Limited Activities because of a Cognitive reason; LimActP_i_ = the i-th total number of Limited Activities because of a Physical reason; ICFscoreActi = the performance score given to the i-th limited activity; ICFscoreActC_i_ = the performance score given to the i-th limited activity with a Cognitive reason of limitation; ICFscoreActP_i_ = the performance score given to the i-th limited activity with a Physical reason of limitation; TNA*4 = Maximal disability; ${\sum^{\text{​}}}_{\text{i}=0}^{\text{NAlim}}\;\text{LimAc}t_{i}*\text{ICFscoreAc}t_{i}$ = absolute disability; $\;{\sum^{\text{​}}}_{\text{i}=0}^{\text{NAlim}}\;\text{LimAct}C_{i}*\text{ICFscoreAct}C_{i}$ = absolute disability because of cognitive reasons; $\;{\sum^{\text{​}}}_{\text{i}=0}^{\text{NAlim}}\;\text{LimAct}P_{i}*\text{ICFscoreAct}P_{i}$ = absolute disability because of physical reasons.

A “global disability index” (DI) can be calculated for b-ADL (b-ADL-DI) and i-ADL (i-ADL-DI) by taking into account a maximal disability (TNA multiplied by ICFScoreAct 4, which is equal to complete difficulty) and an absolute disability (LimAct multiplied by the severity of each limitation [ICFScoreAct]) (see Fig 1). Furthermore, for each reason of limitation, an index can be calculated. In this study, a “cognitive disability index” (CDI) and a “physical disability index” (PDI) for both b-ADL (b-ADL-CDI and b-ADL-PDI) and i-ADL (i-ADL-CDI and i-ADL-PDI) is computed, considering exclusively activities that are limited because of respectively cognitive and physical limitations (see Fig 1). When limitations are caused by multiple reasons (e.g., using transportation is limited to both physical and cognitive reasons), reasons can be assigned in both indices (e.g., i-ADL-CDI and i-ADL-PDI). All indices are expressed as percentages, with higher scores representing more disability.

Example (for b-ADL): A person previously performed 6 b-ADL (TNA = 6). ICFScore 0 is assigned to four activities, ICFScore 1 is assigned to one activity because of cognitive problems, and ICFScore 3 is assigned to one activity because of physical factors. This person has two limited activities, and the maximal disability is 24 (TNA*4). His b-ADL-DI is 16.6% (all limited activities are taken into account), the b-ADL-CDI is 4.2% (only the activities limited because of cognitive reasons are taken into account), and the b-ADL-PDI is 12.4% (only the activities limited because of physical reasons are taken into account).

### Statistical analyses to determine the clinimetric properties

From the diagnostic procedure, data regarding the MMSE, Katz Index, Lawton Scale, number of comorbidities, and medication use were extracted for this study. There were no missing data. Statistical analyses were performed with IBM SPSS for Mac (version 22.0) (SPSS Inc, Illinois, United States) with an α-level set two sided at *p* < 0.05 for all analysis. Demographic and clinical characteristics (i.e., age, education, gender, number of used medications, and number of comorbidities) and the MMSE, Katz Index, Lawton Scale were evaluated between groups by one-way ANOVA with Bonferonni post hoc tests or chi-square analysis. The construct validity was checked, in absence of a true golden standard, by determining the new evaluation’s ability to distinguish between HC, MCI, and AD. We hypothesised HC would show less disability than MCI and the latter less than AD, and that the CDI for both b- and i-ADL would differ more than DI and PDI. The indices for b- and i-ADL were compared across the groups using analysis of covariance (ANCOVA) in which age, number of used medications, number of comorbidities, level of education, and gender were included in the model as covariates. Secondly, in checking the construct validity, we calculated correlations between the indices and the MMSE. We hypothesised that (1) the CDI for both b- and i-ADL would show higher correlations with the MMSE than the DI and PDI since the CDI expresses solely deficits caused by cognitive disorders and (2) the i-ADL-CDI would show higher correlations than b-ADL-CDI because performing i-ADL is more vulnerable to cognitive disorders. Correlation analyses were performed using Pearson correlation between the MMSE, Katz Index, Lawton Scale, and the indices. To interpret the correlations, the guideline by Evans (1996) [88] was used: .00–.19 very weak, .20–.39 weak, .40–.59 moderate, .60–.79 strong, and .80–1.0 excellent. The interrater reliability was checked by comparing simultaneous observation of the interview by two independent raters in a sample of 25 participants and was evaluated by computing intraclass correlation coefficients (ICC) in a two-way mixed model and 95% confidence intervals (CI). Lastly, discriminative validity was evaluated by calculating receiver-operating-characteristics (ROC) curves and the AUC with cutoffs, sensitivity, and specificity for the new evaluation of b-ADL and i-ADL and for the Katz Index and Lawton Scale. The ROC curves and AUC for the new evaluation were compared with the Katz Index and Lawton Scale to determine the added diagnostic value of the new evaluation by using the method of DeLong et al. (1988) [89] in MedCalc (version 14.8.1.0) (MedCalc Software, Mariakerke, Belgium).

## Results

### Participants’ characteristics

All participants reported to have enjoyed the assessment. The interviews lasted between 8 and 15 min for both b- and i-ADL; no adverse events occurred during the diagnostic procedure or the ICF-based evaluation of b- and i-ADL. Table 5 shows the demographic and clinical characteristics of the participants. In comparison to HC, patient groups had less years of education (F(2,220) = 13.7, *p* < .001) and reported more comorbidities (F(2,220) = 20.1, *p* < .001) and use of medications (F(2,220) = 15.3, *p* < .001). Between MCI and AD, no significant differences were found for age, education, medication use, and comorbidities. For MCI and AD, data about their everyday functioning were obtained by spouses (40.3%), children (50.0%), or close friends (9.7%). Almost half of them (48.6%) lived together with the person with MCI or AD. No significant differences between MCI and AD were found for relationship of the proxy (χ^2^(3) = 4.64, *p* < .199) and whether or not living together with the proxy (χ^2^(1) = 1.37, *p* < .241).

**Table 5:** Participants' characteristics.

	HC	MCI	AD	*p*-value^a^	Post-hoc *p*-value^b^		
	(*n* = 79)	(*n* = 73)	(*n* = 71)		HC versus MCI	MCI versus AD	HC versus AD
Age							
Mean (±SD)	76.6 (±6.6)	80.8 (±5.0)	81.9 (±5.4)	<0.001	<0.001	0.788	<0.001
Range	65.0–91.0	71.0–91.0	71.0–93.0				
Gender							
Female, *n* (%)	57.0	57.5	67.6	0.352			
Education, yrs							
Mean (±SD)	13.3 (±3.1)	11.4 (±2.8)	10.9 (±2.7)	<0.001	<0.001	0.989	<0.001
Range	6–18	6–18	6–18				
Medication Use							
Mean (±SD)	4.0 (±2.5)	5.9 (±2.9)	6.4 (±2.9)	<0.001	<0.001	0.884	<0.001
Range	0–10	1–12	1–12				
Comorbidities							
Mean (±SD)	3.3 (±2.0)	5.0 (±2.5)	5.8 (±2.6)	<0.001	<0.001	0.179	<0.001
Range	0–9	1–12	1–12				
MMSE							
Mean (±SD)	28.6 (±1.2)	26.1 (±2.0)	21.7 (±2.8)	<0.001	<0.001	<0.001	<0.001
Range	26–30	22–30	16–28				
b-ADL Katz Index (./24)							
Mean (±SD)	6.2 (±0.6)	7.4 (±1.8)	8.1 (±2.6)	<0.001	0.001	0.049	<0.001
Range	6–10	6–13	6–20				
i-ADL Lawton & Brody Scale (./27)							
Mean (±SD)	24.4 (±2.8)	20.2 (±4.2)	16.9 (±4.0)	<0.001	<0.001	<0.001	<0.001
Range	15–27	10–27	9–26				

HC: healthy comparison; MCI: mild cognitive impairment; AD: Alzheimer disease; MMSE: Mini-Mental State Examination; SD: standard deviation. b-ADL according to the Katz Index has a minimum score of 6/24; lower scores are indicating higher autonomy. i-ADL according to the Lawton & Brody Scale has a minimum score of 9/27; lower scores are indicating lower autonomy.

^a^ χ^2^ for categorical variables and one-way ANOVA for continuous variables.

^b^ Differences between groups were evaluated with Bonferonni post hoc tests.

### Construct validity

#### Indices of everyday functioning in i-ADL

For i-ADL, participants performed an average of eight activities (minimum 4—maximum 9; SD ± 1.2). There were no significant differences between the groups on TACT i-ADL [F(2, 215) = 0.01, *p* = 0.987]. As illustrated in Table 6, the i-ADL-DI and i-ADL-CDI expressed significantly more severe deficits in AD than in MCI and in MCI than in HC (F(2,215) = 61.5, *p* < .001; F(2,215) = 88.9, *p* < .001). The i-ADL-CDI had a moderate correlation with the MMSE (r = –0.588, 95% CI [–0.697 to –0.482]; *p* < .001). The i-ADL-PDI expressed no significant differences between the groups and had a weak correlation with the MMSE (r = –0.348, 95% CI [–0.473 to –0.224]; *p* < .001).

**Table 6:** Construct validity—Indices of everyday functioning in b- and i-ADL.

	HC	MCI	AD	Post- hoc P-value ^a^		
	(*n* = 79)	(*n* = 73)	(*n* = 71)	HC < MCI	MCI < AD	HC < AD
i-ADL-DI, %				<0.001	<0.001	<0.001
Mean (±SD)	11.2% (±11.6)	36.8% (±22.3)	55.6% (±20.7)			
Range	0.0–79.1	0.0–94.4	5.5–100			
i-ADL-CDI, %				<0.001	<0.001	<0.001
Mean (±SD)	1.3% (±4.7)	19.3% (±18.6)	43.2% (±21.9)			
Range	0.0–38.5	0.0–94.4	2.7–93.7			
i-ADL-PDI, %				0.393	1.000	0.146
Mean (±SD)	6.1% (±10.7)	17.2% (±16.5)	20.7% (±19.6)			
Range	0.0–79.1	0.0–64.8	0.0–83.3			
b-ADL-DI, %				0.031	0.011	<0.001
Mean (±SD)	2.0% (±4.8)	10.9% (±12.9)	17.5% (±15.8)			
Range	0.0–25.0	0.0–55.0	0.0–75.0			
b-ADL-CDI, %				0.119	<0.001	<0.001
Mean (±SD)	0% (±0.0)	2.1% (±5.8)	6.3% (±9.3)			
Range	0.0–0.0	0.0–29.1	0.0–46.8			
b-ADL-PDI, %				0.263	0.518	0.016
Mean (±SD)	1.9% (±4.5)	9.1% (±11.0)	12.6% (±15.1)			
Range	0.0–25.0	0.0–40.0	0.0–75.1			

HC: healthy comparison; MCI: mild cognitive impairment; AD: Alzheimer disease; i-ADL-DI: instrumental Activities of Daily Living—Disability Index; i-ADL-CDI: instrumental Activities of Daily Living—Cognitive Disability Index; i-ADL-PDI: instrumental Activities of Daily Living—Physical Disability Index; b-ADL-DI: basic Activities of Daily Living—Disability Index; b-ADL-CDI: basic Activities of Daily Living—Cognitive Disability Index; b-ADL-PDI: basic Activities of Daily Living—Physical Disability Index; SD: standard deviation.

^a^ Differences between groups were evaluated by one-way ANCOVA with Bonferonni post hoc test.

#### Indices of everyday functioning in b-ADL

For b-ADL, participants always performed all activities. Persons with AD and MCI expressed significantly more severe deficits in b-ADL-DI than HC (F(2,215) = 12.6, *p* = < .05) (Table 6). The b-ADL-CDI showed significantly more severe deficits in AD than HC and MCI (F(2,215) = 17.3, *p* < .001). The b-ADL-PDI showed significantly more severe deficits in AD than HC (F(2,215) = 3.9, *p* = .016). No significant differences were found between HC and MCI or MCI and AD. All indices of b-ADL had a weak correlation with the MMSE (ranging from –0.316 to –0.411; all *p* < .001).

### Interrater reliability

The interrater reliability (*n* = 25) was excellent for b-ADL-DI (ICC = .965, 95% CI [.920–.984]); b-ADL-CDI (ICC = .943, 95% CI [.872–.975]); b-ADL-PDI (ICC = .934, 95% CI [.850–.971]); i-ADL-DI (ICC = .986, 95% CI [.968–.994]); i-ADL-CDI (ICC = .986, 95% CI [.969–.994]); and i-ADL-PDI (ICC = .972, 95% CI [.973–.988]) (all *p* < .001). No significant differences between raters were observed.

### Discriminative validity

Table 7 present the results of the ROC curves for the Katz Index, Lawton Scale, and the indices of the new evaluation.

**Table 7:** Discriminative validity of the b- and i-ADL indices between HC, MCI, and AD.

HC versus MCI						
	AUC (S.E.)	95% CI	*p*-value^a^	Optimal Cutoff	Sensitivity	Specificity
Lawton & Brody Scale (Total)	0.797 (0.03)	0.726–0.868		22.5	67.6%	79.5%
i-ADL-DI	0.858 (0.03)	0.793–0.922	0.042	17.2%	80.8%	84.8%
i-ADL-CDI	0.895 (0.02)	0.840–0.950	0.002	3.8%	80.8%	91.1%
i-ADL-PDI	0.724 (0.04)	0.642–0.805	0.038	7.7%	65.8%	67.1%
Katz Index (Total)	0.709 (0.04)	0.629–0.796		6.5	52.7%	87.3%
b-ADL-DI	0.736 (0.04)	0.655–0.818	0.255	6.2%	53.4%	89.6%
b-ADL-CDI	0.568 (0.04)	0.477–0.660	<0.001	2.1%	13.7%	100%
b-ADL-PDI	0.711 (0.04)	0.628–0.795	0.945	2.1%	58.9%	77.2%
MCI versus AD						
	AUC (S.E.)	95% CI	*p*-value^a^	Optimal Cutoff	Sensitivity	Specificity
Lawton & Brody Scale (Total)	0.719 (0.04)	0.633–0.799		18.5	64.8%	69.3%
i-ADL-DI	0.736 (0.04)	0.654–0.818	0.349	44.1%	70.4%	74.3%
i-ADL-CDI	0.805 (0.03)	0.734–0.876	0.010	23.6%	74.6%	70.3%
i-ADL-PDI	0.546 (0.04)	0.452–0.641	<0.001	8.8%	66.2%	41.1%
Katz Index (Total)	0.593 (0.04)	0.499–0.649		8.5	32.4%	78.1%
b-ADL-DI	0.631 (0.04)	0.539–0.722	0.099	18.3%	46.5%	80.8%
b-ADL-CDI	0.648 (0.04)	0.558–0.739	0.210	6.2%	36.6%	87.7%
b-ADL-PDI	0.555 (0.04)	0.460–0.649	0.197	10.4%	45.1%	64.4%
HC versus AD						
	AUC (S.E.)	95% CI	*p*-value^a^	Optimal Cutoff	Sensitivity	Specificity
Lawton & Brody Scale (Total)	0.932 (0.01)	0.895–0.970		20.5	77.5%	88.5%
i-ADL-DI	0.968 (0.01)	0.938–0.998	0.010	30.5%	90.1%	96.2%
i-ADL-CDI	0.990 (0.00)	0.978–1.000	<0.001	10.5%	93.0%	97.5%
i-ADL-PDI	0.774 (0.03)	0.698–0.849	<0.001	7.7%	71.8%	67.1%
Katz Index (Total)	0.787 (0.03)	0.711–0.864		7.5	53.5%	94.9%
b-ADL-DI	0.828 (0.03)	0.759–0.897	0.039	10.4%	60.6%	93.7%
b-ADL-CDI	0.718 (0.04)	0.633–0.803	0.054	2.1%	43.7%	100%
b-ADL-PDI	0.748 (0.04)	0.668–0.829	0.155	10.4%	45.1%	94.0%

b-ADL-DI: basic Activities of Daily Living—Disability Index; b-ADL-CDI: basic Activities of Daily Living—Cognitive Disability Index; b-ADL-PDI: basic Activities of Daily Living—Physical Disability Index; i-ADL-DI: instrumental Activities of Daily Living—Disability Index; i-ADL-CDI: instrumental Activities of Daily Living—Cognitive Disability Index; i-ADL-PDI: instrumental Activities of Daily Living—Physical Disability Index.

^*a*^*p*-values indicate the significant difference between the indices and the total scores of the Katz Index and Lawton Scale. Differences were calculated by comparing ROC curves with the method of DeLong et al. (1988) [89].

#### Indices of everyday functioning in i-ADL

The AUC of i-ADL-DI ranges from 0.736 to 0.968 and has a significantly better discriminative accuracy than the Lawton Scale for differentiating between HC and MCI and between HC and AD with DeLong’s test (all *p* < .05). The i-ADL-CDI showed best accuracy, expressed by AUCs ranging from 0.805 to 0.968 and a significantly higher discriminative power than the Lawton Scale with DeLong’s test (all *p* < .05). The i-ADL-PDI did not show a better accuracy than the Lawton Scale.

#### Indices of everyday functioning in b-ADL

The AUC of b-ADL-DI showed with DeLong’s test (*p* < .05) a significantly better discriminative accuracy than the Katz Index in differentiating between HC and AD with an AUC of 0.828. The b-ADL-CDI and b-ADL-PDI showed no better diagnostic accuracy.

## Discussion

This study developed and validated an evaluation of everyday functioning in b- and i-ADL by (1) adopting the activities of the Katz Index and Lawton Scale and linking them to the definitions and codes of the ICF, (2) by developing a scoring system based on the performance qualifiers of the ICF, and (3) by adding the possibility to take causes of limitations in performance into account. This new evaluation takes the person as his or her own reference. By doing so, it is possible to compute a set of indices. This study determined the construct validity, discriminative validity, and interrater reliability of this new evaluation in a geriatric population.

The new evaluation showed more accuracy in evaluating b- and i-ADL compared to the Katz Index and Lawton scale and subsequently has the potential to improve diagnostic differentiation between HC, mild NCD (e.g., MCI), and major NCD (e.g., AD). As hypothesised, this evaluation followed the hierarchical continuum of functional decline [19]; b-ADL-DI and i-ADL-DI showed significantly less disability in HC than in MCI and the latter less than in AD. The i-ADL-DI showed more disability than b-ADL-DI and had a significantly better accuracy than the Lawton Scale to differentiate HC from MCI and AD. The b-ADL-DI, in its turn, had a significantly better accuracy than the Katz Index in differentiating HC from AD. Other promising results were seen in the i-ADL-CDI. Although the original Lawton Scale cannot detect mildly affected quality of performance in i-ADL [90], the i-ADL-CDI could detect subtle functional deficits of persons with MCI and AD. The i-ADL-CDI demonstrated a significantly better accuracy than the Lawton Scale and is able to distuinguish between HC, MCI, and AD. This illustrates that it is important to make a distinction in causes, especially in older patients in whom physical limitations are commonly seen and also affect everyday functioning. When considering the diagnosis of NCD, it is of utmost importance to determine to what extent functional limitation is due to cognitive limitations and not due to other causes [17]. The b-ADL-CDI showed, as hypothesized, significantly more severe deficits in AD than in HC and MCI but had no better accuracy than the Katz Index. This can be explained by the fact that performing b-ADL is less vulnerable for cognitive decline and is often largely spared until later stages of the disease (i.e., moderate or severe dementia) [91]. If limitations are observed in MCI or mild AD, it will rather be caused by other reasons such as physical limitations, as illustrated in the b-ADL-PDI. The b-ADL-PDI showed significantly more severe limitations in AD than in HC and had similar accuracy to the Katz Index. Although the differentiation between normal cognition and mild AD is usually not much of a diagnostic dilemma in clinical context, the results of this study clearly state that b-ADL distinguish well when reasons for limitations are taken into account.

Until now, self- and informant-report scales did not show sound psychometrical properties [9,43] and were not considered as the best methods to evaluate everyday functioning since they might over- and underestimate functional ability [32,92]. There is growing evidence that performance-based evaluations might have more advantages over other assessment approaches [9,32,33]. However, only few of them are developed to assess MCI or mild dementia [55,93], and they are mostly too time- and cost-consuming to be administered [9,31]. Two recent performance-based instruments, the Erlangen Test of Activities of Daily Living in Mild Dementia or Mild Cognitive Impairment (ETAM) [55] and the Sydney Test of Activities of Daily Living in Memory Disorders (STAM) [94] have been developed with the aim to assess everyday activities in a time-efficient and reliable way for persons with MCI or mild dementia. Both evaluations show good psychometric characteristics, are easy to administer, and seem to be valuable in clinical practice and research. However, our i-ADL-DI and i-ADL-CDI show similar accurate validity to discriminate between HC, MCI, and AD. So, although this study developed a report-based measure—which may not be as accurate in detecting functional difficulties in persons with mild cognitive decline—the results of this study indicate that the ICF-based evaluation of b- and i-ADL might compete with the recently developed performance-based tools as the standard for classifying functional status and decline [32,33,55,94]. Ongoing research will clarify this and is already assessing the convergent validity between the ICF-based evaluation of b- and i-ADL and a performance-based measure.

Although many studies have already tried to improve the use of the Katz Index and the Lawton Scale, not all improvements were relevant for the diagnosis of cognitive disorders. In this study, we attempted to achieve more clarity, transparency, and nuance by maintaining the activities of the Katz Index and the Lawton Scale but by adopting the terminology and the scoring system of the ICF. A first advantage is that each activity is clearly defined by definitions according to the ICF. In contrast to the content of the original Katz Index and Lawton Scale—which varies depending on setting and circumstances [58,95]—the ICF definitions provide clear descriptions of the content of activities. In this way, no more doubt can arise about the exact content of activities such as, e.g., doing laundry (should ironing also be considered?) or using transportation (should driving a car also be considered?). Furthermore, since the ICF definitions do not impose a manner of performing, this evaluation will remain useful for future generations and might also have advantages across cultures since b- and i-ADL will always be applicable. Secondly, this evaluation only considers activities that are relevant for a person. In contrast with other scales, activities that are gender-dependent or a person has never performed in his or her life are not be taken into account. In this way, each person is considered as his or her own reference and is compared to his or her own previous level of everyday functioning, as suggested by Ganguli (2013) [12]. This might also be considered as an advantage for use in other generations and cultures. Thirdly, by using the detailed ICF qualifiers—ranging from 0 to 4—this evaluation provides a more sensitive scoring system, as recommended by Jekel et al. (2015) [9]. This new evaluation makes it possible to calculate indices and showed an excellent interrater reliability in this study. Lastly, this evaluation has the advantage to discriminate between reasons of limitations. Although other tools such as the FAQ [48], ETUQ [49], and ECog [50] are also valuable instruments in assessing individuals with NCD, they do not make a distinction between reasons of functional decline.

Although the results of our study are promising and may imply a change in the evaluation of everyday functioning in clinical practice, some considerations need to be made. First, a measurement bias might have occurred by using different methods of reporting ADL in HC and patients with MCI and AD. Although a report-based method has the clinical advantages of being easy to obtain, minimally disturbing, and of low cost, proxy and patient-based measures can be biased by mood status, social desirability, diminished awareness, denial, and other cognitive deficits [96,97]. But since informant-reports are generally preferred to self-report in evaluating everyday functioning in clinical practice and research settings, a reliable proxy was questioned about the everyday functioning of participants with MCI and AD in this study [97]. This closely resembles clinical reality, in which health care professionals have to work with the information that is available. Nevertheless, we could not rule out that the informants were not mildly cognitively impaired themselves. However, 50.0% of the informants were children of the persons with MCI and AD and had an estimated age range of 45 to 65 y. Although it is known that children of persons with AD are at high risk of cognitive disorders as they age, it seems unlikely that this would have influenced the results at this point of time. For the HC, only self-report was used because prior research in cognitively healthy older persons suggested that self-report evaluations are generally accurate indicators of ADL for older persons who demonstrate insight into their functional abilities [96,98]. Additionally, a second reflection must be made about the participants in this study. The patients with AD and MCI represent a clinically relevant sample but were significantly older, had more comorbidities, and took more medications than the HC. This suggests that the patient groups were frailer and might have experienced more functional problems. However, not all medications and comorbidities would be expected to contribute equally to functional impairment. Furthermore, this study did not report any measures of current depressive symptoms. The presence of a major depression was ruled out prior to the diagnosis in MCI and AD. However, mild to moderate depressive symptoms are an important comorbidity of cognitive disorders and may have an impact on everyday activities [99–102].

As a result, the contrast between groups might be larger than would be expected in a clear clinical sample. However, in the statistical analysis, our data was controlled for possible confounders such as age, medication use, number of comorbidities, level of education, and gender. Lastly, another consideration is that HC were—apparently—cognitively healthy persons. Yet, it is still possible that mild cognitive problems were present in some of them. However, we used strict cutoffs of MMSE—which can be considered as a valuable instrument for cognitive screening—in order to rule out cognitive deficits.

Based on the results of this study, we argue that this evaluation can contribute to the diagnostic differentiation between cognitively healthy ageing, mild NCD (e.g., MCI), and major NCD (e.g., AD). Particularly, the i-ADL-CDI might be useful. Since it is likely that decline in everyday functioning occurs over time, and this change leads to a conversion from mild to major NCD, further research—a longitudinal prospective follow up study—should address the predictive validity of this evaluation as follow-up assessment [14].

In conclusion, this new ICF-based evaluation for b- and i-ADL addresses important issues in assessing everyday functioning by (1) providing an operationalization of the evaluated activities by ICF codes and definitions, (2) providing a detailed scoring system that is based on the ICF qualifiers, and (3) by making a differentiation in causes of limitations. With validation in longitudinal prospective cohorts, this evaluation might offer a useful addition to the common diagnostic process and be of added value in a multidisciplinary approach with established cognitive and mood measures and biomarkers.

## Supporting information

S1 Dataset Dataset indices. (XLSX)

S2 Dataset Dataset interrater indices. (XLSX)

S1 STARD Checklist STARD checklist. (DOCX)

## Abbreviations

AD: Alzheimer disease
ADL: activities of daily living
AUC: area under the curve
CDI: cognitive disability index
DI: disability index
ECog: Everyday Cognition
ETAM: Erlangen Test of Activities of Daily Living in Mild Dementia or Mild Cognitive Impairment
ETUQ: Everyday Technology Use Questionnaire
FAQ: Functional Activities Questionnaire
HC: healthy comparison
ICC: intraclass correlation coefficient
ICF: International Classification of Human Functioning, Disability and Health
MCI: mild cognitive impairment
MMSE: Mini-Mental State Examination
NCD: neurocognitive disorder
PDI: physical disability index
STAM: Sydney Test of Activities of Daily Living in Memory Disorders
