# Supplementary material for: Early diagnosis of mild cognitive impairment and mild dementia through basic and instrumental activities of daily living: Development of a new evaluation tool
Source: PLoS Med. 2017 Mar 14;14(3):e1002250. doi: 10.1371/journal.pmed.1002250 (PMC5349421; doi:10.1371/journal.pmed.1002250)
Supplement: S1 STARD Checklist — (DOCX) [file pmed.1002250.s003.docx]

|  | **Section & Topic** | **No** | **Item** | **Reported on page #** |
| --- | --- | --- | --- | --- |
|  |  |  |  |  |
|  | **TITLE OR ABSTRACT** |  |  |  |
|  |  | **1** | Identification as a study of diagnostic accuracy using at least one measure of accuracy  (such as sensitivity, specificity, predictive values, or AUC  Early diagnosis of mild cognitive impairment and mild dementia through basic and instrumental activities of daily living: development of a new evaluation tool. | Title page |
|  | **ABSTRACT** |  |  |  |
|  |  | **2** | Structured summary of study design, methods, results, and conclusions  (for specific guidance, see STARD for Abstracts)  **Background**  Assessment of activities of daily living (ADL) is paramount to determine impairment in everyday functioning and to ensure accurate early diagnosis of neurocognitive disorders. Unfortunately, most common ADL-tools are limited in their use in a diagnostic process. This study developed a new evaluation by adopting the items of the Katz-Index (basic (b-) ADL) and Lawton Scale (instrumental (i-) ADL), defining them with the terminology of the International Classification of Human Functioning, Disability and Health (ICF), adding the scoring system of the ICF and adding the possibility to identify underlying causes of limitations in ADL.  **Methods and findings**  The construct validity, inter-rater reliability and discriminative validity of this new evaluation were determined. From 2015 until 2016, older persons (65 – 93yrs) with normal cognitive ageing (HC) (n=79), Mild Cognitive Impairment (MCI) (n=73) and Alzheimer’s disease (AD) (n=71) underwent a diagnostic procedure for neurocognitive disorders at the geriatric day hospital of the Universitair Ziekenhuis Brussel (Brussels, Belgium). Additionally, the ICF-based evaluation for b- and i-ADL was carried out. A global Disability Index (DI), a Cognitive DI (CDI) and a Physical DI (PDI) were calculated. The i-ADL-CDI showed high accuracy and higher discriminative power than the Lawton Scale in differentiating HC and MCI (AUC=0.895, 95% CI .840 - .950, *p*=.002), MCI and AD (AUC=0.805, 95% CI .805 - .734, *p*=.010) and HC and AD (AUC=0.990, 95% CI .978 – 1.000, *p*<.001). The b-ADL-DI showed significantly better discriminative accuracy than the Katz-Index in differentiating HC and AD (AUC=0.828, 95% CI .759 - .897, *p*=.039). This study was conducted in a clinically relevant sample. However, heterogeneity between HC, MCI and AD and the use of different methods of reporting ADL might limit this study.  **Conclusions**  This evaluation of b- and i-ADL can contribute to the diagnostic differentiation between cognitively healthy ageing and neurocognitive disorders in older age. This evaluation provides more clarity and nuance in assessing everyday functioning by using an ICF-based terminology and scoring system. Also the possibility to take underlying causes of limitations into account seems to be valuable since it is crucial to determine the extent to which cognitive decline is responsible for functional impairment in diagnosing neurocognitive disorders. Particular the i-ADL-CDI might be useful in clinical practice since it determines impairment in i-ADL exclusively due to cognitive limitations. Although, further validation of this evaluation is necessary. | Abstract |
|  | **INTRODUCTION** |  |  |  |
|  |  | **3** | Scientific and clinical background, including the intended use and clinical role of the index test  Currently, the most commonly used tools for assessing b- and i-ADL are respectively the Katz-Index (22) and the Lawton Scale (57) (31, 58, 59). Although in widespread use, both scales have the shortcomings as mentioned above: they have poorly described psychometric properties, the scoring systems are not sensitive enough to detect subtle deficits and they do not identify causes of limitations in ADL (9, 43, 59-62). Many studies have attempted to improve the potential use of the Katz-Index and Lawton Scale, including using item response theory methods (34), providing short versions of these scales (63) or by combining both scales in new evaluations (64, 65). However, these improvements could not overcome all mentioned shortcomings.  Therefore, this study set out to develop a new tool to evaluate b- and i-ADL for diagnostic purposes in a geriatric population with NCD. This evaluation is based on the International Classification of Functioning, Disability and Health (ICF), developed by the World Health Organization (WHO) (66). The ICF provides a framework for describing everyday functioning and advances the understanding and measurement of disability (67). It is increasingly being applied in clinical practice and research, and has gained acceptance as the worldwide framework of assessing human functioning (68, 69). The new evaluation adopted the activities of the Katz-Index and Lawton Scale – since they are considered sound as items for describing functioning in b- and i-ADL (70) – and were defined with the ICF-terminology. Beside, the new evaluation took over the scoring system of the ICF and added the possibility to determine underlying causes of limitations. This evaluation might be used in clinical and research settings to evaluate everyday functioning in NCD since it has an advance over currently used report-based scales by applying the ICF-terminology and scoring system. | Introduction, paragraph 5 |
|  |  | **4** | Study objectives and hypotheses  In this study, the construct validity, inter-rater reliability and discriminative validity of this new evaluation were determined. We hypothesized that the ICF-based evaluation of b- and i-ADL will have a good construct validity, inter-rater reliability and will be able to discriminate between cognitively healthy persons (HC), MCI and AD. | Introduction, paragraph 6 |
|  | **METHODS** |  |  |  |
|  | *Study design* | **5** | Whether data collection was planned before the index test and reference standard  were performed (prospective study) or after (retrospective study)  After the procedure, at the same day, trained occupational therapists carried out the new ICF-based evaluation of everyday functioning in b- and i-ADL. | Methods, participants and procedure, paragraph 6 |
|  | *Participants* | **6** | Eligibility criteria  *Cognitively healthy persons*  Exclusion criteria for the HC (n=79) were a history of NCD, a score <26/30 on the MMSE and any self- or informant-based complaint of functional or cognitive deficits, which were suggestive of MCI or AD. Exclusion criteria were a score of <80/105 on the total CamCog score, <18/27 on the memory section of the CamCog score and <8/12 on the MIS and VAT (82, 83).  *Patients with MCI*  Patients with MCI (n=73) were diagnosed by clinical consensus of the multidisciplinary team and fulfilled the diagnostic criteria for a-MCI as defined by Petersen (5). The presence of a major depression was ruled out prior to the diagnosis of MCI.  *Patients with AD*  Patients with AD (n=71) fulfilled the National Institute for Neurological and Communicative Disorders and Stroke - Alzheimer’s Disease and Related Disorder Association (NINDS-ADRDA) criteria (84). Decisions on the diagnosis of AD were also based on the results of the diagnostic procedure and were carefully taken by consensus of the multidisciplinary team. When the presence of a major depression was presumed, this was ruled out prior to the diagnosis of AD. | Methods, participants and procedure, paragraph 2 - 4 |
|  |  | **7** | On what basis potentially eligible participants were identified  (such as symptoms, results from previous tests, inclusion in registry)  Three groups of community-dwelling older persons (≥65yrs) were recruited consecutively through the geriatric day hospital of an academic teaching hospital (UZ Brussel, Belgium): (1) HC, (2) patients with MCI and (3) with Alzheimer’s disease (AD). Patients with MCI and AD underwent a procedure for the diagnosis of cognitive disorders, performed by a multidisciplinary team and considered as good clinical practice (72). This procedure consisted of a physical and neurological examination, clinical history-taking and neuropsychological assessment using Mini-Mental State Examination (MMSE) (73), Cambridge Examination for mental disorders of the elderly, cognitive part (CamCog) (74), Alzheimer’s Disease Assessment Scale, cognitive subscale (75), Visual Association Test (VAT) (76), Memory Impairment Screen (MIS) (77), Trail Making Test, part A and B (78, 79), Frontal Assessment Battery (80) and Geriatric Depression Scale (GDS-15) (81). The procedure was completed by an evaluation of ADL using the Katz-Index and Lawton Scale, an extensive laboratory blood testing and imaging of the brain by CT or MRI scan. HC were recruited separately from the diagnostic process for MCI and AD. They represent a heterogeneous sample of community-dwelling volunteers and geriatric patients who visited the geriatric day hospital for the diagnosis or treatment of conditions other than cognitive disorders (e.g. osteoporosis). HC were evaluated by the researchers using the same neuropsychological assessment and evaluation of ADL as MCI and AD. For all groups, the number of co-morbidities and medication use were inventoried | Methods, participants and procedure, paragraph 1 |
|  |  | **8** | Where and when potentially eligible participants were identified (setting, location and dates)  Three groups of community-dwelling older persons (≥65yrs) were recruited consecutively through the geriatric day hospital of an academic teaching hospital (UZ Brussel, Belgium). | Methods, participants and procedure, paragraph 1 |
|  |  | **9** | Whether participants formed a consecutive, random or convenience series  Three groups of community-dwelling older persons (≥65yrs) were recruited consecutively through the geriatric day hospital of an academic teaching hospital (UZ Brussel, Belgium). | Methods, participants and procedure, paragraph 1 |
|  | *Test methods* | **10a** | Index test, in sufficient detail to allow replication  **The ICF-based evaluation of everyday functioning in b- and i-ADL**  This evaluation has been designed as a semi-structured interview that takes 10 minutes to complete. For the HC, self-report was used. For MCI and AD, proxy-report was conducted.  **Items according to the ICF definitions**  According to the linking rules of Cieza et al. (2005) (85), the content of each item included in the Katz-Index (22) and the Lawton Scale (57, 86) was linked to one or more definitions of the activities component of the ICF (Table 1a + 1b).  **Interview protocol**  First, the participant or proxy is asked whether an activity was performed during the past years. It is expected that the interviewer uses the ICF-definitions to clarify the content of an activity. Each activity is rated for its relevance which means that it is currently performed or it was previously performed by the individual. If activities have not been carried out during the past years because they were not relevant for an individual, they are not taken into account. This is mainly important for i-ADL, since these activities may never have been performed before (e.g. gender relevant) and are consequently irrelevant for the individual. For b-ADL all items are relevant for every individual, since – according to the definition of Reuben (87) - these activities are necessary to survive. The sum of relevant activities leads to the Total Number of relevant Activities (TNA). There is no cut-off of how many items are allowed to be not relevant.  **Scoring system**  The participant or proxy is asked how the activities are currently being performed. Based on the description, the investigator assigns a score. The scoring system adopted the performance qualifiers of the ICF, consisting of a five-point scale ranging from 0 (no difficulty to perform) to 4 (complete difficulty or unable to perform) (Table 2). Each score describes how an activity is performed and reflects the degree of autonomy (ICFScoreAct). The qualifiers were operationalized based on the experience of this research team with the development of the advanced ADL tool (a-ADL tool) (35, 88) and on a previous qualitative study (89). The sum of activities with a limitation (score ≥1) leads to the total number of Limited Activities (LimAct).  **Causes of limitations**  If a score of 1 or higher is assigned, the interviewer determines the underlying cause of limitation by asking the participant what causes the limitations. The interviewer probes following questions: “Why do you/does (s)he performs this activity differently?” or “What causes the need for help to perform this activity?”. In this way, the interviewer interprets the story of the participant and can distinguish cognitive reasons (e.g. global mental functions, memory, attention…), physical reasons (e.g. sensorial functions, mobility, stability…), intra-personal reasons (e.g. switch in field of interest), social reasons (e.g. loss of partner) and environmental reasons (e.g. car sold, moving to a new place, …) of limitations. The assignment of a reason is dichotomous: “yes” when a reason is present and “no” when a reason is absent. It is possible to assign more than one reason of limitation.  **Indices**  A 'global disability index' (DI) can be calculated for b-ADL (b-ADL-DI) and i-ADL (i-ADL-DI) by taking into account a maximal disability (TNA multiplied by ICFScoreAct 4, which is equal to complete difficulty) and an absolute disability (LimAct multiplied by the severity of each limitation (ICFScoreAct) (see figure 1). Furthermore, for each reason of limitation an index can be calculated. In this study, a ‘cognitive disability index’ (CDI) and a ‘physical disability index’ (PDI) for both b-ADL (b-ADL-CDI and b-ADL-PDI) and i-ADL (i-ADL-CDI and i-ADL-PDI) is computed, considering exclusively activities that are limited because of respectively cognitive and physical limitations (see figure 1). When limitations are caused by multiple reasons (e.g. using transportation is limited to both physical and cognitive reasons), reasons can be assigned in both indices (e.g. i-ADL-CDI and i-ADL-PDI). All indices are expressed as percentages, with higher scores representing more disability. | Methods, paragraph 7 – 12 |
|  |  | **10b** | Reference standard, in sufficient detail to allow replication  A procedure for the diagnosis of cognitive disorders, performed by a multidisciplinary team and considered as good clinical practice (72). This procedure consisted of a physical and neurological examination, clinical history-taking and neuropsychological assessment using Mini-Mental State Examination (MMSE) (73), Cambridge Examination for mental disorders of the elderly, cognitive part (CamCog) (74), Alzheimer’s Disease Assessment Scale, cognitive subscale (75), Visual Association Test (VAT) (76), Memory Impairment Screen (MIS) (77), Trail Making Test, part A and B (78, 79), Frontal Assessment Battery (80) and Geriatric Depression Scale (GDS-15) (81). The procedure was completed by an evaluation of ADL using the Katz-Index and Lawton Scale, an extensive laboratory blood testing and imaging of the brain by CT or MRI scan. | Methods, participants and procedure, paragraph 1 |
|  |  | **11** | Rationale for choosing the reference standard (if alternatives exist) | N.A. |
|  |  | **12a** | Definition of and rationale for test positivity cut-offs or result categories  of the index test, distinguishing pre-specified from exploratory | N.A. |
|  |  | **12b** | Definition of and rationale for test positivity cut-offs or result categories  of the reference standard, distinguishing pre-specified from exploratory | N.A. |
|  |  | **13a** | Whether clinical information and reference standard results were available  to the performers/readers of the index test  When conducting the new evaluation, the occupational therapists were blinded to the results of the other evaluations or the diagnosis. | Methods, participants and procedure, paragraph 6 |
|  |  | **13b** | Whether clinical information and index test results were available  to the assessors of the reference standard  The ICF-based evaluation of b- and i-ADL was performed after the diagnostic procedure for cognitive disorders, so index test results were not available to the assessors of the reference standard. | Methods, participants and procedure, paragraph 6 |
|  | *Analysis* | **14** | Methods for estimating or comparing measures of diagnostic accuracy  Lastly, discriminative validity was evaluated by calculating receiver-operating-characteristics (ROC) curves and the area under the curve (AUC) with cut-offs, sensitivity and specificity for the new evaluation of b-ADL and i-ADL and for the Katz-Index and Lawton Scale. The ROC curves and AUC for the new evaluation were compared with the Katz-Index and Lawton Scale to determine the added diagnostic value of the new evaluation by using with the method of DeLong et al. (1988) (84) in MedCalc (version 14.8.1.0) (MedCalc Software, Mariakerke, Belgium). | Methods, statistical analyses, paragraph 13 |
|  |  | **15** | How indeterminate index test or reference standard results were handled | N.A. |
|  |  | **16** | How missing data on the index test and reference standard were handled | N.A. |
|  |  | **17** | Any analyses of variability in diagnostic accuracy, distinguishing pre-specified from exploratory | N.A. |
|  |  | **18** | Intended sample size and how it was determined | Not provided |
|  | **RESULTS** |  |  |  |
|  | *Participants* | **19** | Flow of participants, using a diagram | Not provided |
|  |  | **20** | Baseline demographic and clinical characteristics of participants  Table 4 shows the demographic and clinical characteristics of the participants. In comparison to HC, patient groups had less years of education (F(2,220)= 13.7, p<.001) and reported more comorbidities (F(2,220)= 20.1, p<.001) and use of medications (F(2,220)=15.3, p<0.001). Between MCI and AD, no significant differences were found for age, education, medication use and comorbidities. For MCI and AD, data about their everyday functioning was obtained by spouses (40.3%), children (50.0%) or close friends (9.7%). Almost half of them (48.6%) lived together with the person with MCI or AD. No significant differences between MCI and AD were found for relationship of the proxy (χ^2^(3)=4.64, p<.199) and whether or not living together with the proxy (χ^2^(1)=1.37, p<.241). | Results, paragraph 1 |
|  |  | **21a** | Distribution of severity of disease in those with the target condition | N.A. |
|  |  | **21b** | Distribution of alternative diagnoses in those without the target condition | N.A. |
|  |  | **22** | Time interval and any clinical interventions between index test and reference standard  After the procedure, at the same day, trained occupational therapists carried out the new ICF-based evaluation of everyday functioning in b- and i-ADL. | Methods, participants and procedure paragraph 6 |
|  | *Test results* | **23** | Cross tabulation of the index test results (or their distribution) by the results of the reference standard  Table 7 | Results, table 7. |
|  |  | **24** | Estimates of diagnostic accuracy and their precision (such as 95% confidence intervals)  Table 7 and in the results section:  *Indices of everyday functioning in i-ADL*  The AUC of i-ADL-DI ranges from 0.736 to 0.968 and has a significantly better discriminative accuracy than the Lawton Scale for differentiating between HC and MCI, and between HC and AD with De longs test (all p<.05). The i-ADL-CDI showed best accuracy, expressed by AUCs ranging from 0.805 to 0.968 and a significantly higher discriminative power than the Lawton Scale with De Longs test (all p<.05). The i-ADL-PDI did not show a better accuracy than the Lawton Scale.  *Indices of everyday functioning in b-ADL*  The AUC of b-ADL-DI showed with De Longs test (p<.05) a significantly better discriminative accuracy than the Katz-Index in differentiating between HC and AD with an AUC of 0.828. The b-ADL-CDI and b-ADL-PDI showed no better diagnostic accuracy. | Results, table 7 |
|  |  | **25** | Any adverse events from performing the index test or the reference standard  All participants reported to have enjoyed the assessment. No adverse events occurred during the diagnostic procedure or the ICF-based evaluation of b- and i-ADL. | Results, paragraph 1 |
|  | **DISCUSSION** |  |  |  |
|  |  | **26** | Study limitations, including sources of potential bias, statistical uncertainty, and generalizability  Although the results of our study are promising and may imply a change in the evaluation of everyday functioning in clinical practice, some considerations need to be made. First, a measurement bias might have occurred by using different methods of reporting ADL in HC and patients with MCI and AD. Although a report-based method has the clinical advantages that it is easy to obtain, minimally disturbing and low cost, proxy and patient-based measures can be biased by mood status, social desirability, diminished awareness, denial and other cognitive deficits (99, 100). But since informant-reports are generally preferred to self-report in evaluating everyday functioning in clinical practice and research settings, a reliable proxy was questioned about the everyday functioning of participants with MCI and AD in this study (100). This closely resembles clinical reality, where health care professionals have to work with the information that is available. Nevertheless, we could not rule out that the informants were not mildly cognitively impaired themselves. However, 50.0% of the informants were children of the persons with MCI and AD. Consequently, they can not be considered as having a high risk of cognitive disorders. For the HC, only self-report was used because prior research in cognitively healthy older persons suggested that self-report evaluations are generally accurate indicators of ADL for older persons who demonstrate insight into their functional abilities (99, 101). Additionally, a second reflection must be made about the participants in this study. The patients with AD and MCI represent a clinically relevant sample but were significantly older, had more co-morbidities and took more medications than the HC. This suggests that the patient groups were frailer and might have experienced more functional problems. However, not all medications and co-morbidities would be expected to contribute equally to functional impairment. Furthermore, this study did not report any measures of current depressive symptoms. However the presence of a major depression was ruled out prior to the diagnosis in MCI and AD, mild to moderate depressive symptoms are an important comorbidity of cognitive disorders and may have an impact on everyday activities (102-105). | Discussion, paragraph 4 |
|  |  | **27** | Implications for practice, including the intended use and clinical role of the index test  Based on the results of this study, we argue that this evaluation can contribute to the diagnostic differentiation between cognitively healthy ageing, mild NCD (e.g. MCI) and major NCD (e.g. AD). Particular the i-ADL-CDI might be useful.  In conclusion, this new ICF-based evaluation for b- and i-ADL addresses important issues in assessing everyday functioning by providing (1) an operationalization of the evaluated activities by ICF-codes and definitions, (2) a detailed scoring system which is based on the ICF-qualifiers and (3) by having the major advantage to make a differentiation in causes of limitations. This might offer a useful addition to the common diagnostic process and can be of added value in a multidisciplinary approach with established cognitive and mood measures and biomarkers. | Discussion, paragraph 7 – 8 |
|  | **OTHER INFORMATION** |  |  |  |
|  |  | **28** | Registration number and name of registry | N.A. |
|  |  | **29** | Where the full study protocol can be accessed | Added in supporting information files |
|  |  | **30** | Sources of funding and other support; role of funders  This study was partly supported by Wetenschappelijk Fonds Willy Gepts, UZ Brussel, Belgium. The funders had no role in study design, data collection and analysis, decision to publish, or preparation of the manuscript. | Added in financial disclosure. |
|  |  |  |  |  |

STARD 2015

### AIM

STARD stands for “Standards for Reporting Diagnostic accuracy studies”. This list of items was developed to contribute to the completeness and transparency of reporting of diagnostic accuracy studies. Authors can use the list to write informative study reports. Editors and peer-reviewers can use it to evaluate whether the information has been included in manuscripts submitted for publication.

### Explanation

A **diagnostic accuracy study** evaluates the ability of one or more medical tests to correctly classify study participants as having a **target condition.** This can be a disease, a disease stage, response or benefit from therapy, or an event or condition in the future. A medical test can be an imaging procedure, a laboratory test, elements from history and physical examination, a combination of these, or any other method for collecting information about the current health status of a patient.

The test whose accuracy is evaluated is called **index test.** A study can evaluate the accuracy of one or more index tests. Evaluating the ability of a medical test to correctly classify patients is typically done by comparing the distribution of the index test results with those of the **reference standard**. The reference standard is the best available method for establishing the presence or absence of the target condition. An accuracy study can rely on one or more reference standards.

If test results are categorized as either positive or negative, the cross tabulation of the index test results against those of the reference standard can be used to estimate the **sensitivity** of the index test (the proportion of participants *with* the target condition who have a positive index test), and its **specificity** (the proportion *without* the target condition who have a negative index test). From this cross tabulation (sometimes referred to as the contingency or “2x2” table), several other accuracy statistics can be estimated, such as the positive and negative **predictive values** of the test. Confidence intervals around estimates of accuracy can then be calculated to quantify the statistical **precision** of the measurements.

If the index test results can take more than two values, categorization of test results as positive or negative requires a **test positivity cut-off**. When multiple such cut-offs can be defined, authors can report a receiver operating characteristic (ROC) curve which graphically represents the combination of sensitivity and specificity for each possible test positivity cut-off. The **area under the ROC curve** informs in a single numerical value about the overall diagnostic accuracy of the index test.

The **intended use** of a medical test can be diagnosis, screening, staging, monitoring, surveillance, prediction or prognosis. The **clinical role** of a test explains its position relative to existing tests in the clinical pathway. A replacement test, for example, replaces an existing test. A triage test is used before an existing test; an add-on test is used after an existing test.

Besides diagnostic accuracy, several other outcomes and statistics may be relevant in the evaluation of medical tests. Medical tests can also be used to classify patients for purposes other than diagnosis, such as staging or prognosis. The STARD list was not explicitly developed for these other outcomes, statistics, and study types, although most STARD items would still apply.

### DEVELOPMENT

This STARD list was released in 2015. The 30 items were identified by an international expert group of methodologists, researchers, and editors. The guiding principle in the development of STARD was to select items that, when reported, would help readers to judge the potential for bias in the study, to appraise the applicability of the study findings and the validity of conclusions and recommendations. The list represents an update of the first version, which was published in 2003.

More information can be found on [http://www.equator-network.org/reporting-guidelines/stard](http://www.equator-network.org/reporting-guidelines/stard/).
